# Data on estimation of health hazards associated with pesticide residues in drinking water

**DOI:** 10.1016/j.dib.2022.107830

**Published:** 2022-01-17

**Authors:** Ibrahim El-Nahhal, Yasser El-Nahhal

**Affiliations:** a Université de Toulon, CS 60584, France; b Department of Earth and Environmental Science Faculty of Science, The Islamic University-Gaza, Palestine

**Keywords:** Pesticide, Health hazards, Reference average, Relative average, Reference standard deviation, Relative standard deviation

## Abstract

The dataset presents the occurrence of 113 pesticide residues (PR) in drinking water samples from 31 counties worldwide and correlates their concentrates with human health. The dataset classifies PRs to four toxicity classes. Class IA (extremely toxic), includes four residues with an LD_50_ value < 5 mg/kg. b. w.; class IB (highly toxic compounds), includes 14 residues with an LD_50_ value in the range of 5-<50 mg/kg b w.); Class II, (moderately toxic) includes 55 residues with an LD_50_ value in the range of 50-<500 mg/kg b w.); Class III, (slightly toxic compounds) includes 17 residues with an LD_50_ value in the range of 500-<2000 mg/kg bw. and class IV (less toxic compound) includes 23 residues with an LD_50_ value > 2000 mg/kg bw. The dataset provides a new statistical method that link all PRs together throughout using reference average (Ref Aver), reference standard deviation (Ref Stdev), country average and country standard deviation to show the statistical variations among them. Furthermore, the dataset calculates hazard indices (HIs) and shows its distribution among 31 countries. Noteworthy, the dataset provides advanced techniques to clean water from PRs. Detailed explanation and discussion of the present dataset can be found in the article entitled “Pesticide residues in drinking water, their potential risk to human health and removal options” under article doi: 10.1016/j.jenvman.2021.113611 (El-Nahhal and El-Nahhal, 2021). To the best of our knowledge, this is the first dataset that describes the use of Ref Aver and Ref Stdev to link the averages of all PRs of countries together to show the differences of occurrence and provides several cleaning options of PRs from drinking water.

**Specifications Table**

**Table:** 

Subject	*Environmental Science*
Specific subject area	*Pesticide contamination in drinking water and human health*
Type of data	Chart Figure
How the data were acquired	The data were acquired by downloading the original articles and collecting the required data. In some cases, the data were used as presented in the original article whereas in many cases the data were cleaned, analyzed and used to calculate the average of each country.
Data format	Raw, Analyzed
Description of data collection	*Sixty-nine articles on pesticides residues (PRs) in drinking water from 31 country worldwide were collected. PRs were summarized according to their frequency of detection, chemical group and function in each country. Concentrations of PRs were used to estimate health hazard, average and standard deviation in each country. A reference average and a reference standard deviation were calculated and used.*
Data source location	*PRs residues were collected from published articles listed in**[1]* Acute reference dose of each PRs were collected from Pesticides Properties Database. Available at: https://sitem.herts.ac.uk/aeru/ppdb/[2]. Guidelines for drinking-water quality were collected from WHO: fourth edition incorporating the first addendum. Geneva: Licence: CC BY-NC-SA 3.0 IGO. http://apps.who.int/iris. [3]. Calculation of HQ and HI were conducted according to US EPA 2000. And El-Nahhal 2020 [4] and [7].
Data accessibility	Repository name: Mendeley Data Data identification number: 10.17632/43br98f865.1 Direct URL to data: https://doi.org/10.17632/43br98f865.1
Related research article	I. El-Nahhal, Y. El-Nahhal, Pesticide residues in drinking water, their potential risk to human health and removal options, J Environ Manage. *299* (2021) 113,611. https://doi.org/10.1016/j.jenvman.2021.113611

**Value of the Data**

• These data provide detailed calculations on health hazards associated with PRs in drinking water.
• It can be used by local authorities, policy makers and researchers to improve the environmental health standards.
• It provides a better understanding in the occurrence of PRs in drinking water in 31 countries.
• It shows an important and useful statistical method for other researchers.
• The figures provide an overview of toxicity classes of PRs and their health hazards.

## 1 Data Description

**Fig. 1:**
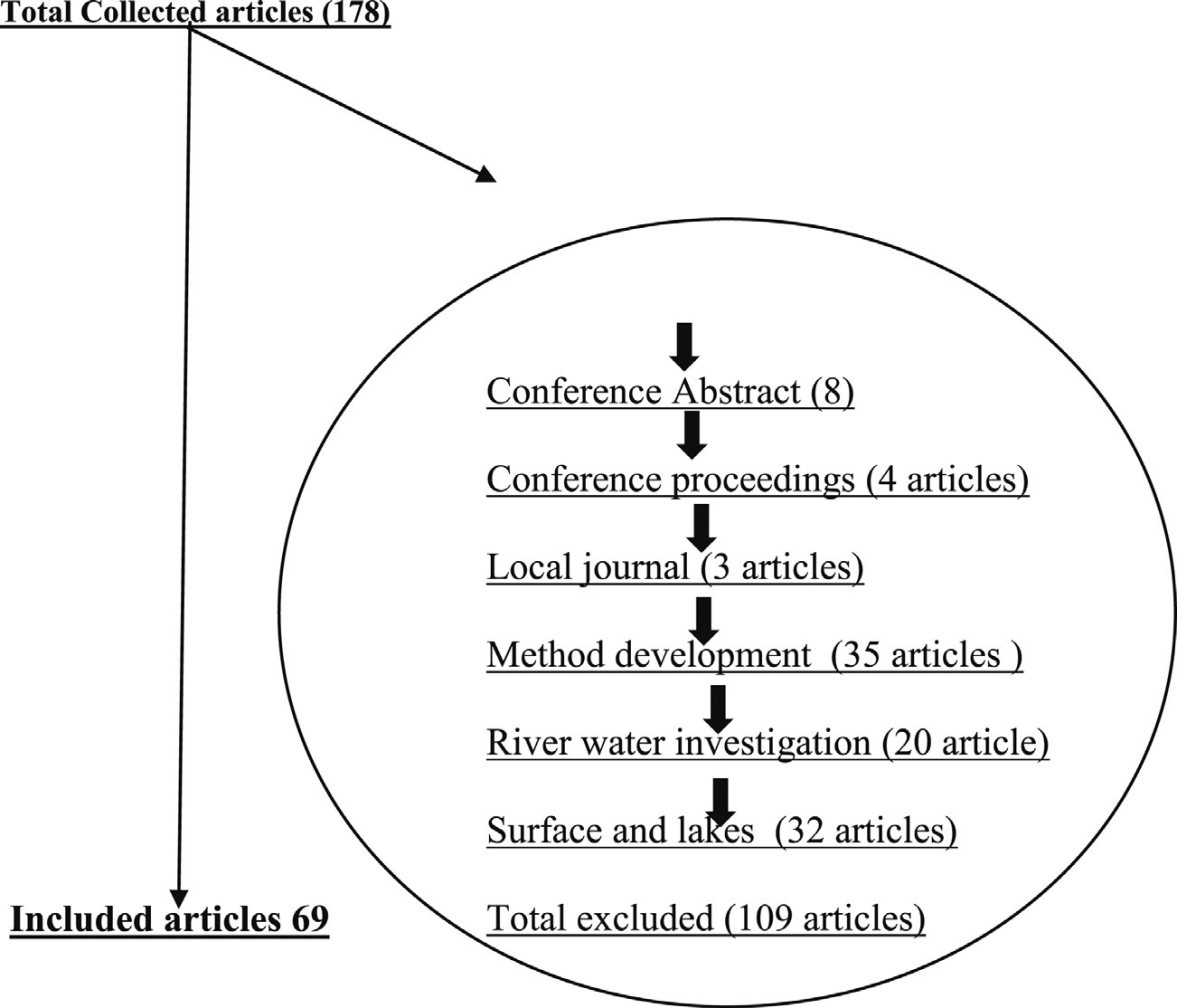
Steps collecting relevant articles, to data collection, screening, sorting, removing and including the relevant articles.

**Fig. 2:**
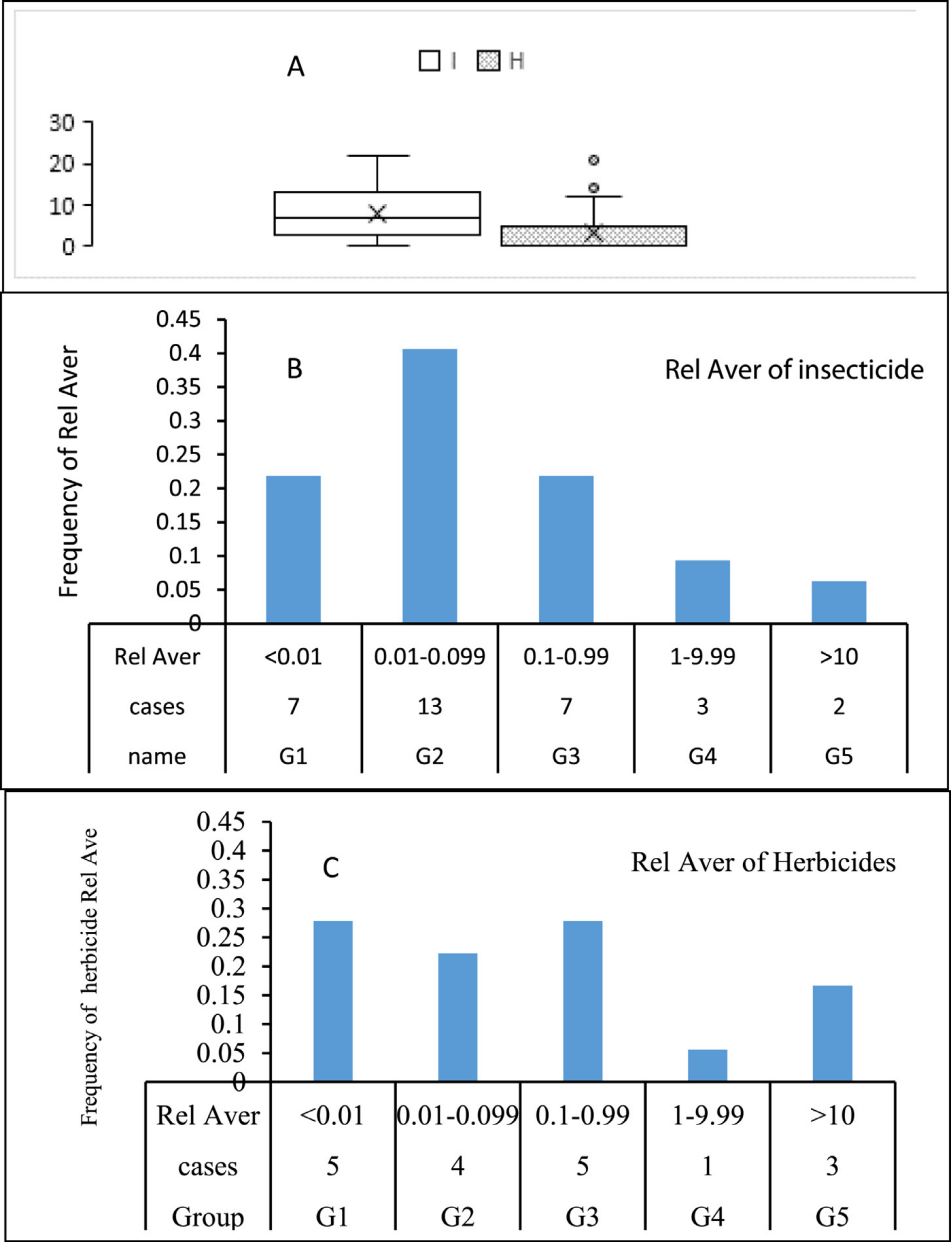
Occurrence of insecticide and herbicide residues in drinking water in several countries worldwide (A); Groups insecticide resides (B) and herbicide residues (C) presented based on Rel. Aver. value as shown on X-axe.

**Fig. 3:**
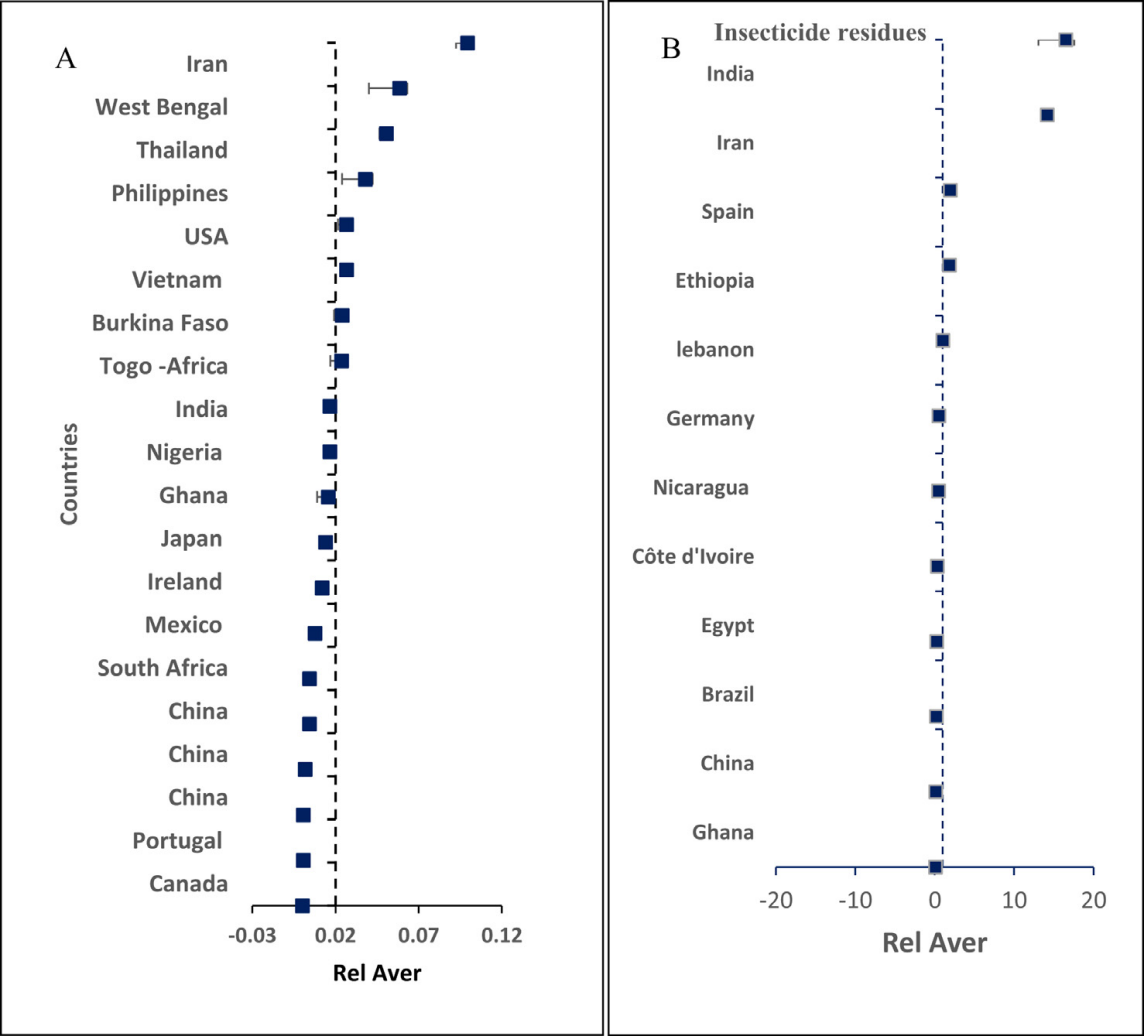
Forest plots of Rel Aver of insecticide residues in drinking water in several countries worldwide. A and B shows countries with having Rel Aver <0.1 and countries with Rel Aver >0.1–20, respectively. Error bars represent relative standard error and relative standard deviation as upper and lower limits to the relative average.

The dataset contains 12 figures describing the study results. For instance, Fig. 1 shows the steps of article collection, excluding irrelevant articles and including the relevant ones. It appears that 61.24% of the collected articles were excluded due to irrelevancy and only 38.76% of article were included. This shows the huge efforts needed to collect the relevant articles from 31 country. Furthermore, the dataset summarize the collected pesticide residues and classifies them to insecticides, herbicides and fungicides (Fig. 2a), and presents insecticide and herbicides residues whereas fungicide residues are not presented in this dataset because they were found only in three countries (i.e. Spain, Brazil and Japan) with low concentrations that do not present health hazards. The dataset classifies the insecticides and herbicide residues according to their relative average (Rel Aver) of concentrations to five groups (Fig. 2b,c). It appears that group 1 (G1), the lowest relative average < 0.1) includes seven countries having insecticide residues and five countries having herbicide concentrations. On the other hand, group 5 (G5) the highest Rel Aver (> 10) includes two and three countries having insecticide residues and herbicide residues, respectively. The other groups (G2–G4) have 23 and 10 countries with insicticides insecticides and herbicide residues, respectively. Fig. 3 shows the distribution of countries in a forest plot. It is obvious that some countries have positions on the left side of the relative averge average (0.01), the dotied dotted line, some others on the dotted line and some on the right side of the dotted line. This indicate the differences among countries. The differences are significantly high between the countries having Rel Aver left side and in right side. Additionally, the countries in contact with the dotted line may have significant differences based on the size of error bars. If the error bars are overlapping together, this indicates no significant differences. If the error bars from left and right side of the dotted line are in contact with the dotted line, this indicate marginal or random differences.

**Fig. 4:**
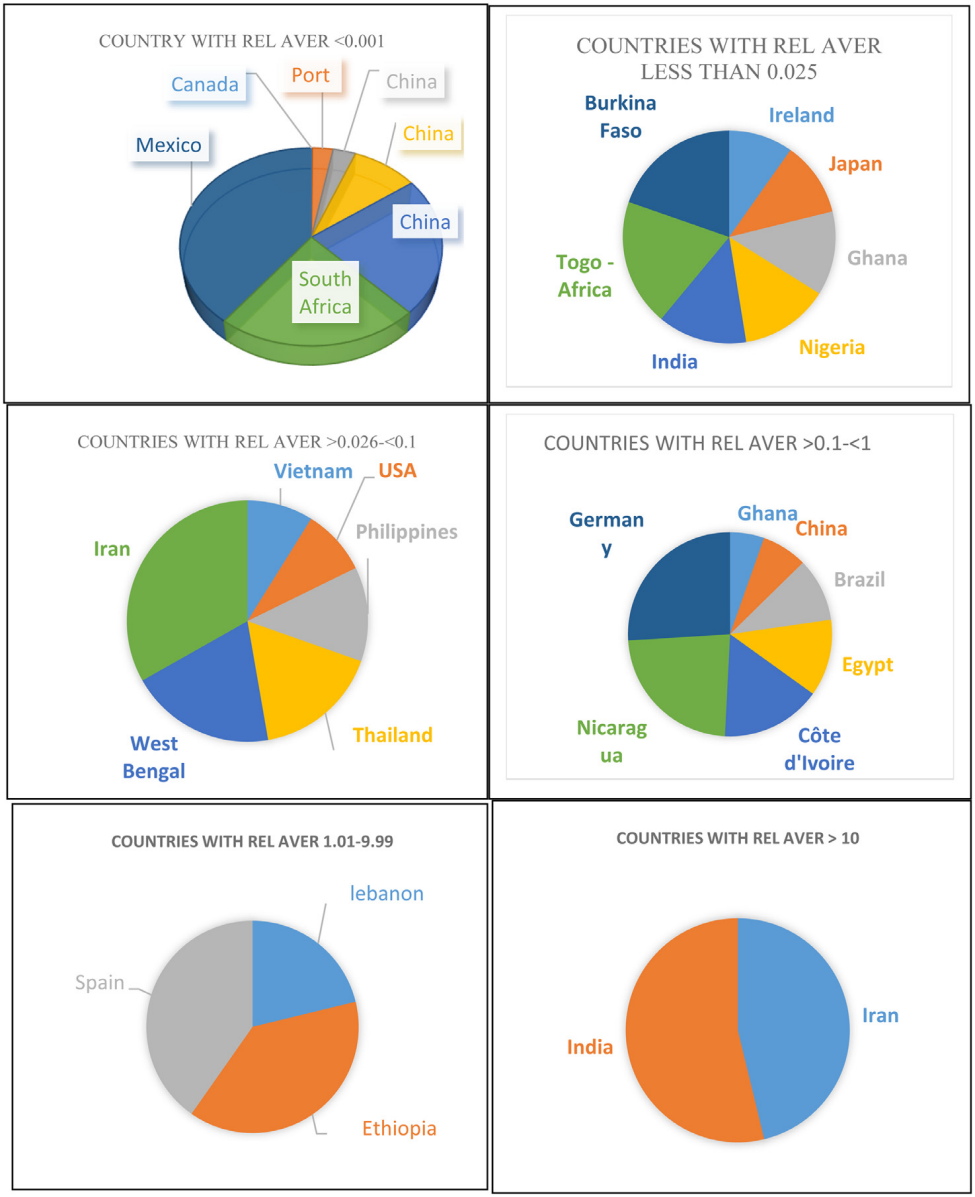
Classes of counties according to their insecticide residues in drinking water samples. Values presented as Rel Aver on insecticide residues in a country.

The occurrence of country Rel Aver of insecticide are shown in Fig. 4. It can be seen that 32 Rel Aver representing 24 countries are presented. The extra number of Rel Aver appeared due to repetition of some countries such as China, India and Iran.

**Fig. 5:**
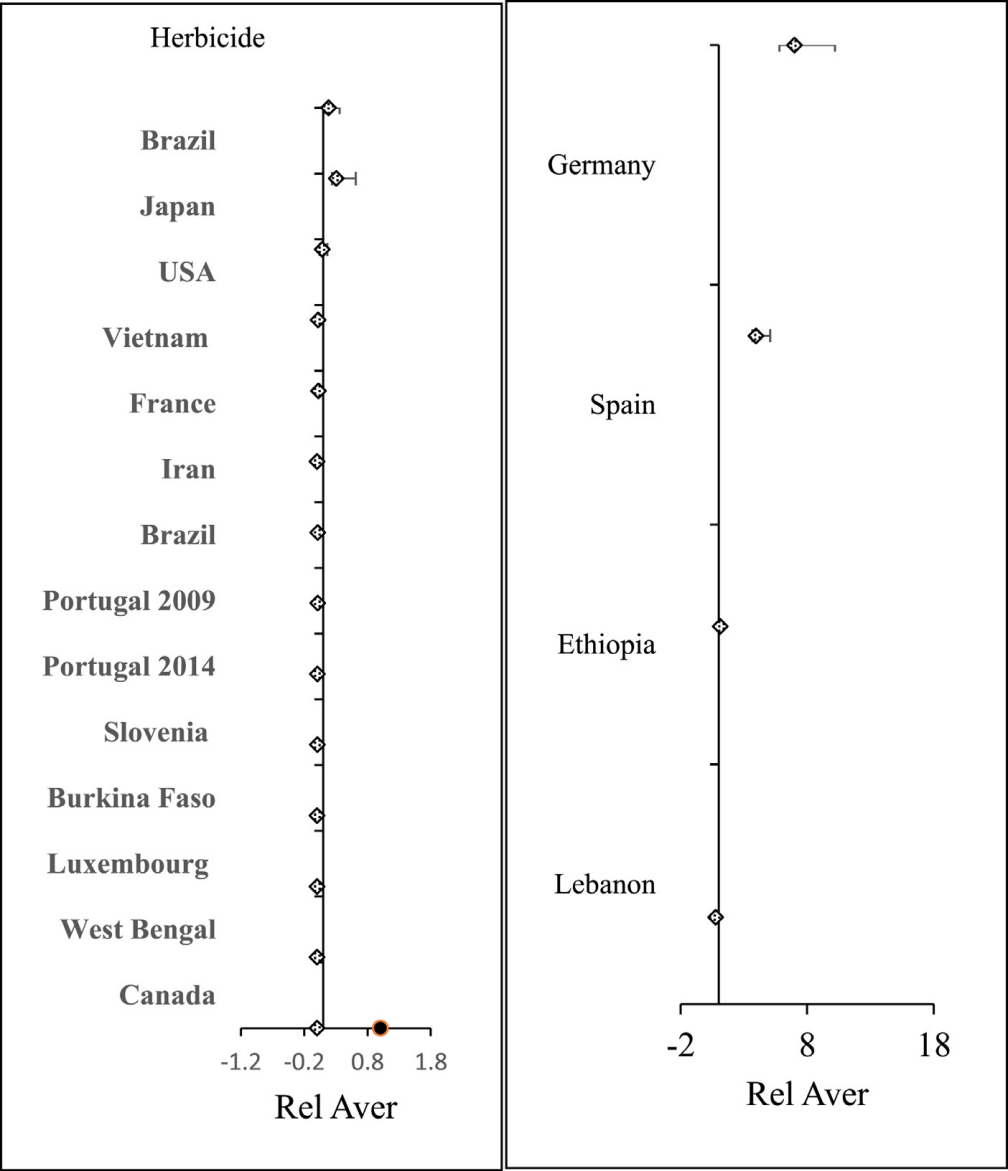
Forest plot of herbicide residues among countries worldwide. Error bars represent Rel Standard deviation and relative standard error as upper and lower limits to the Rel Aver of a country. Left side shows countries have Rel. Aver. range 0.001–0.47 whereas right side shows Rel Aver ≥ 1.

**Fig. 6:**
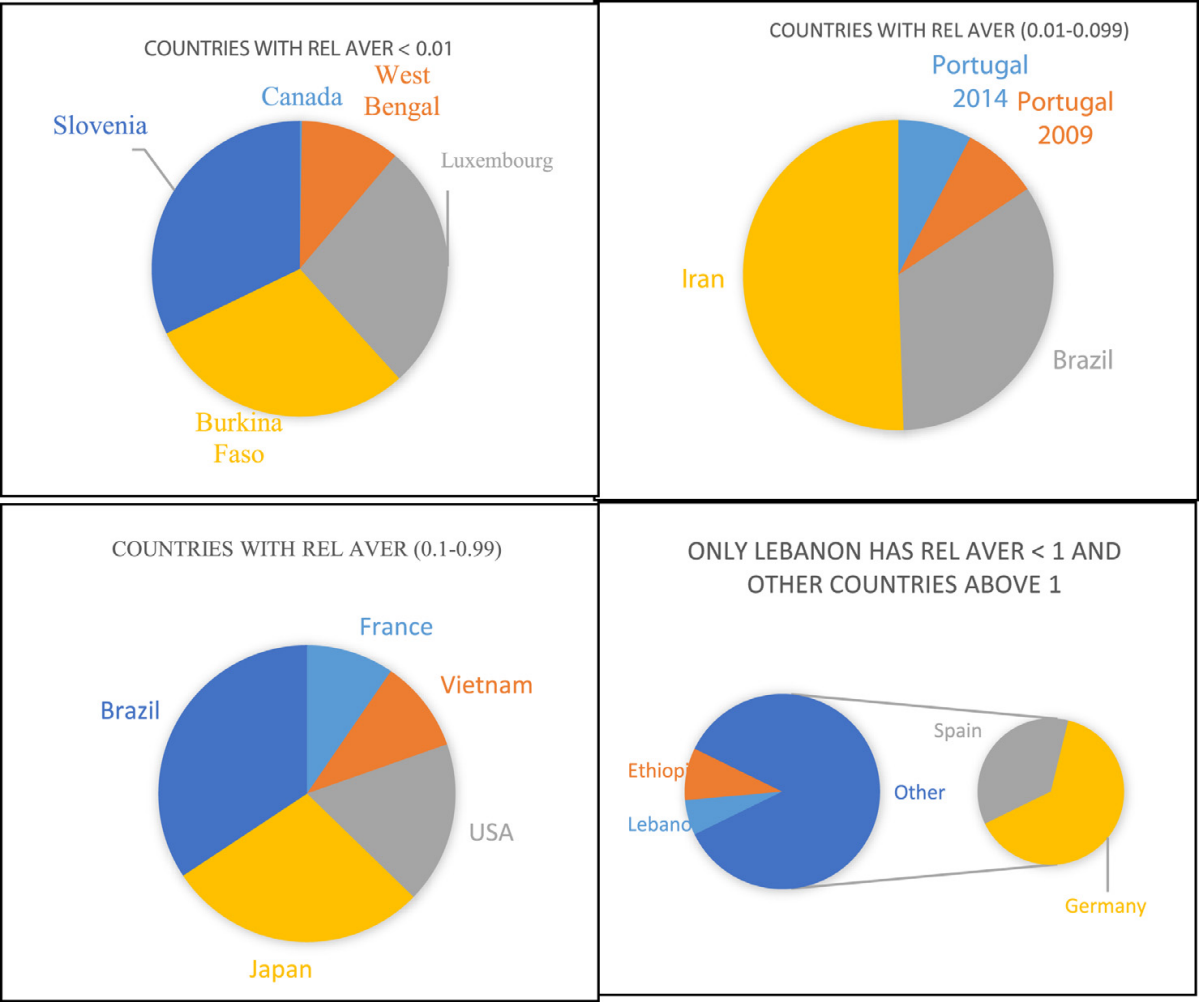
Classes of counties according to their herbicide residues in drinking water samples. Values presented as Rel Aver of herbicide residues in a country.

Fig. 5 shows the distribution of herbicide relative average of several countries worldwide as forest plot. The explanation of these results is similar to those given for Rel Aver of insecticides (Fig. 3). Furthermore, the occurrence of country Rel Aver of herbicide are shown in Fig. 6. It can be seen that 18 Rel Aver of herbicide representing 17 countries worldwide are presented. The difference between the number of Ref Aver and number of countries appeared due to repetition of some countries such as Portugal.

**Fig. 7:**
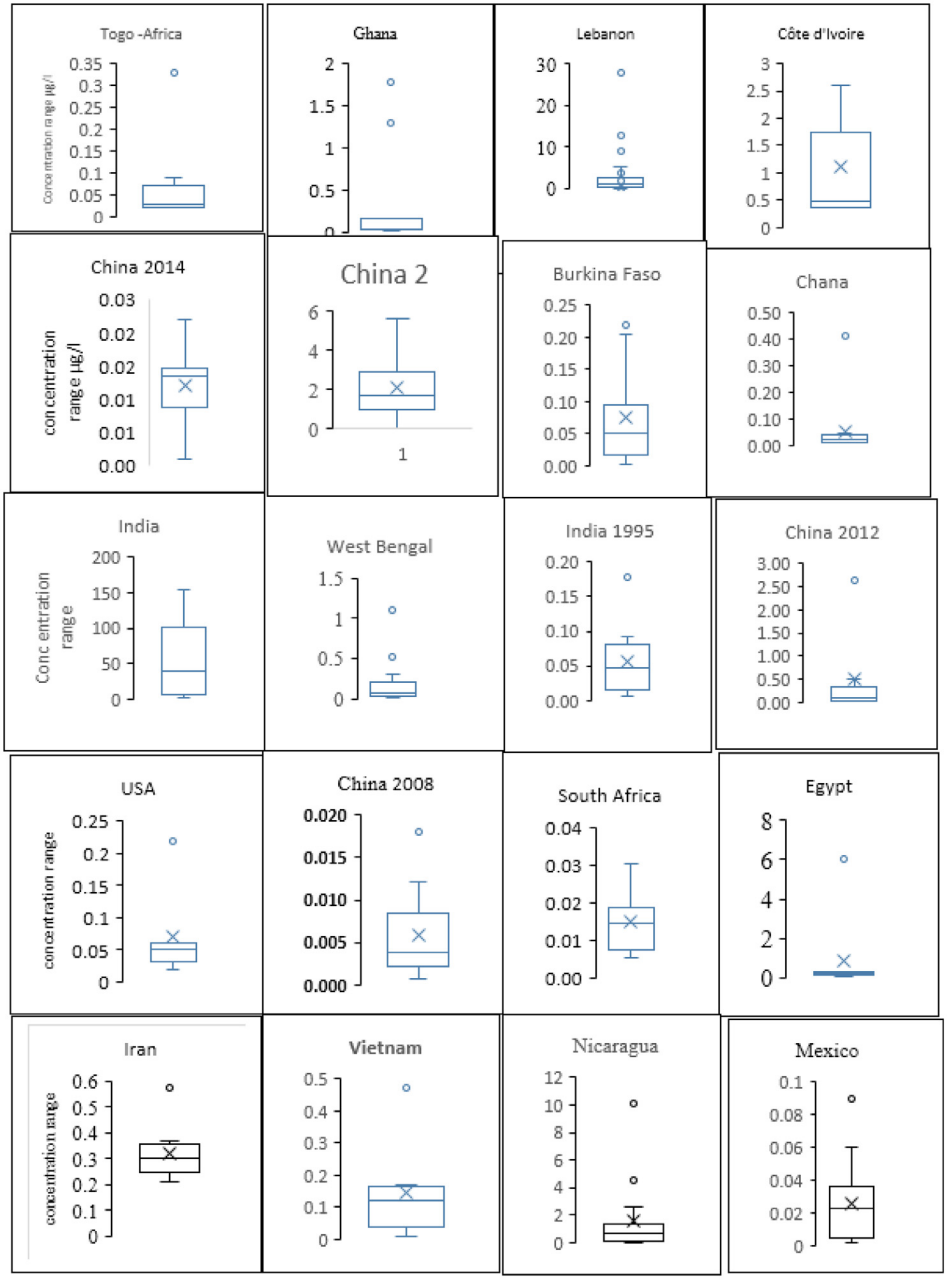
Box plot shows concentration range of IR, minimum concentration, 1st quartile, median, 3rd quartile, and maximum concentrations. These values are denoted by bottom whisker, 1st 2nd and 3rd lines of the box and the upper whisker, respectively. Additionally, x mark inside the box and circle above whisker denote the country average and outliers of insecticide concentration.

**Fig. 8:**
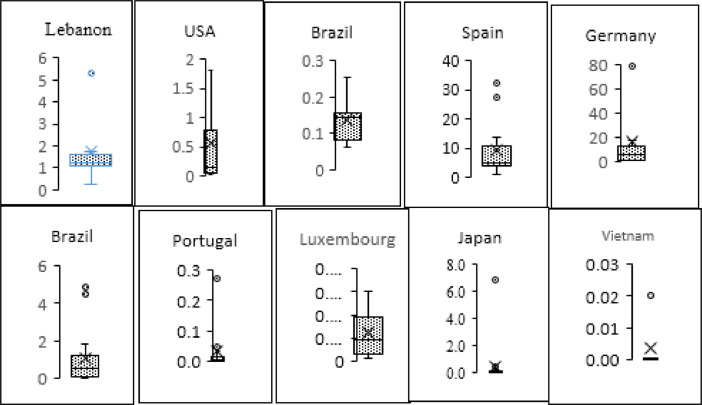
Occurrence of HRs in drinking water samples from several countries. Box plot shows concentration range of HRs, minimum concentration, 1st quartile, median, 3rd quartile, and maximum concentration. These values are denoted by bottom whisker, 1st 2nd and 3rd lines of the box and the upper whisker, respectively. Additionally, x mark inside the box and circle above whisker denote the country average and outliers of insecticide concentration.

Fig. 7 shows the occurrence of insecticide residues as box plot in 20 countries having at least five insecticide residues in drinking. Four countries are not presented here because they have less than five insecticide residues, the essential parameters required to present box plot. It is obvious that the boxes have different sizes and different whiskers indicating different distributions. Additionally, the majority of countries have a high insecticide concentration. This was shown as outliers either low or high. Explanation of the calculation is shown in Materials and methods section. Similarly, Fig. 8 shows the occurrence of HRs in drinking water samples from several countries. Box plot in Figs. 7 and 8 show concentration range of insecticide/herbicide, minimum concentration, 1st quartile, median, 3rd quartile, and maximum concentration. These values are denoted by bottom whisker, 1st 2nd and 3rd lines of the box and the upper whisker, respectively. Additionally, x mark inside a box and circle above whisker denote the country average and outliers of insecticide and/or herbicide concentration.

**Fig. 9:**
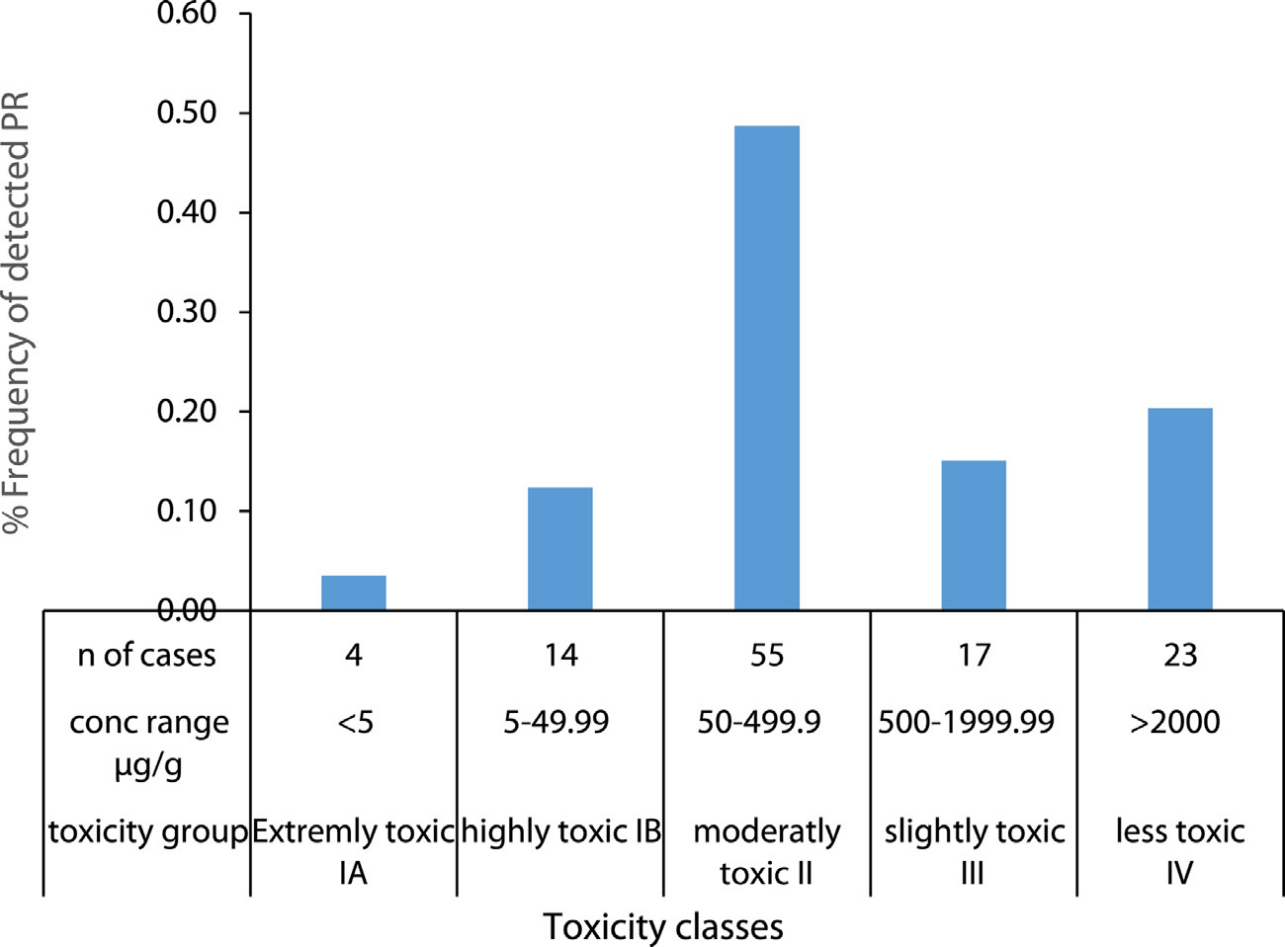
Occurrence of pesticide toxicity classes in drinking water samples collected in 31 countries worldwide.

Fig. 9 shows the occurrence of the toxicity classes of 113 pesticide residues found in drinking water samples collected in 31 countries worldwide. It can be seen that five classes of toxicity are found. For instance, Class IA extremely toxic class (LD_50_ < 5 µg/g) represented by four cases. This class represents less that 5% of all residues. Class IB, highly toxic residues (LD_50_ in the range of 5–49.99 µg/g), represented by 14 residues and occupies 14% of all residues. Class II, moderately toxic class (LD_50_ in the range of 50–499.9 µg/g), represented by 55 residues and occupies 49% of all residues. Class III slightly toxic residues (LD_50_ in the range of 500–1999.9 µg/g), represented by 17 residues and occupies 15% of all residues. Class IV, less toxic residues (LD_50_ > 2000 µg/g) represented by 23 residues and occupies 20% of all residues.

**Fig. 10:**
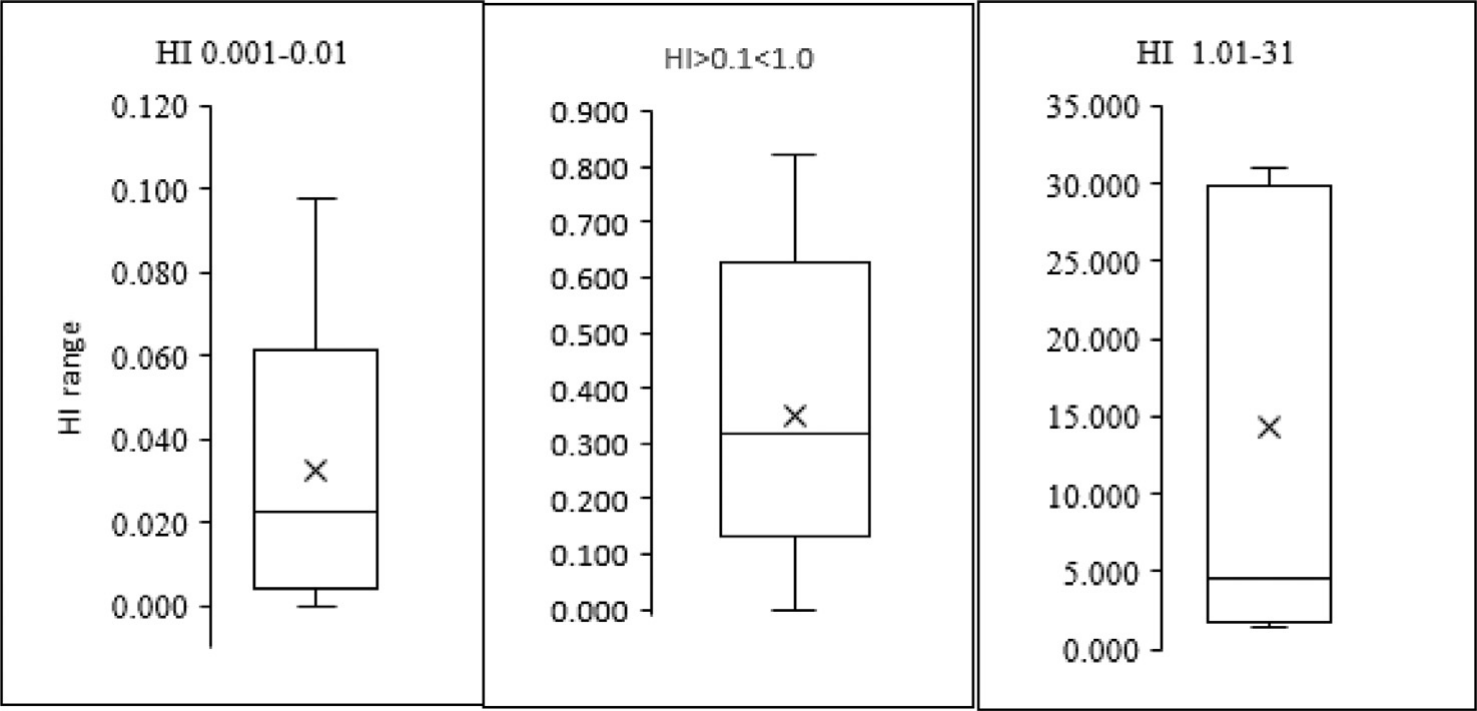
Occurrence of hazard index (HI) associated with IRs in drinking water among several countries. X mark inside the box denotes HI average in the corresponding group.

Hazard index of insecticide residues are shown in Fig. 10. In fact, three categories are shown, HI 0.001–0.01, this is represented by 17 HI from 17 different countries. 0.1 < HI < 1.0, represents HI from 10 countries whereas HI 1.01–31 represent HI from 7 countries worldwide.

It is obvious that the distribution of HI in each category is different as shown by the five parameters of box plot presented in each category. (details of box plot categories are shown above and in the methodology section).

**Fig. 11:**
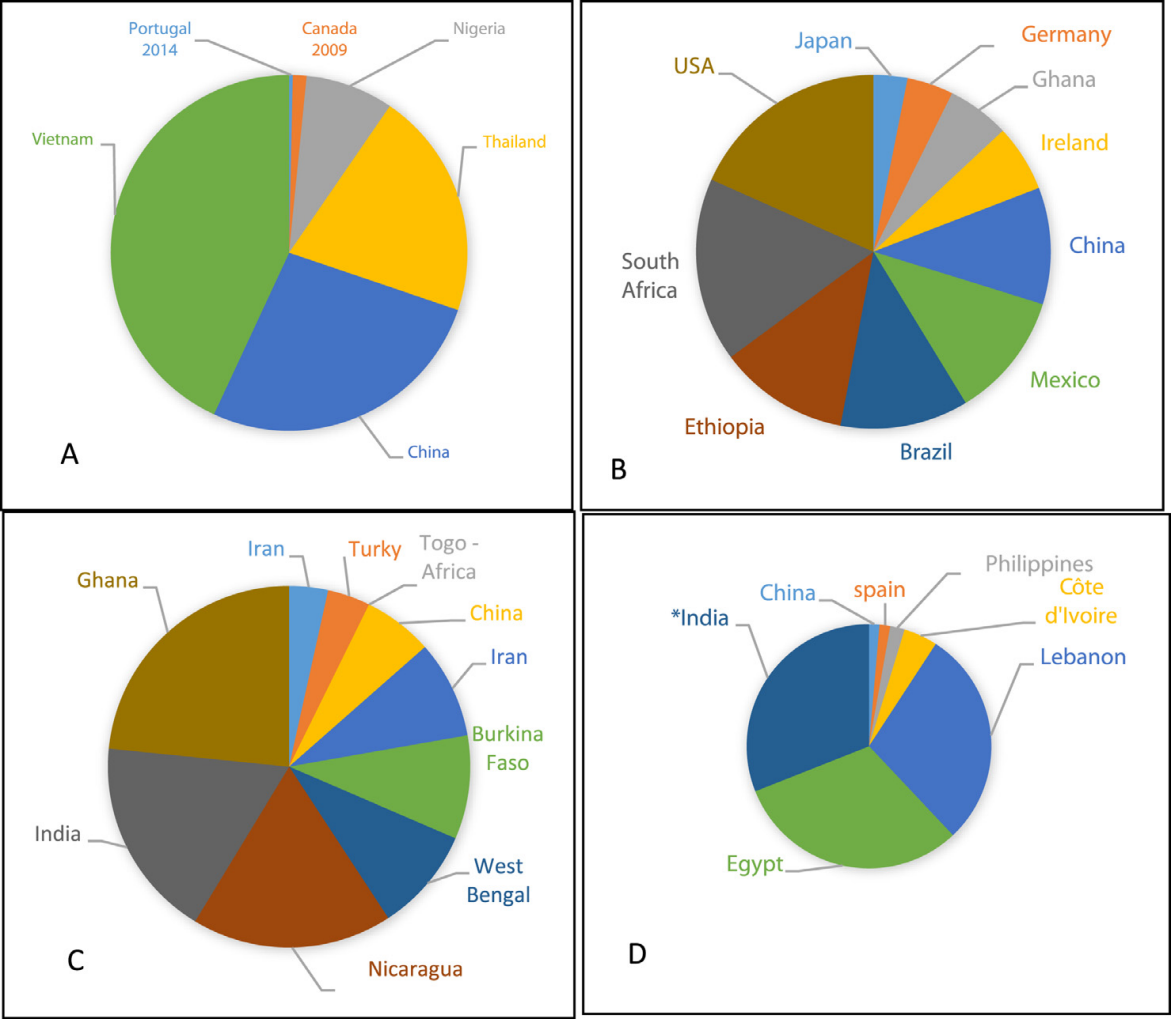
Distribution of countries according to hazard index (HI) associated with IRs in drinking water. A, B, C and D are HI range of: 0001–0.01; 0.011–0.099; 0.1–1.0 and 1.01–30.97, respectively. * indicates 4.5 times higher than presented.

The occurrence of countries in each category of HI are shown in Fig. 11. It can be seen that Fig. 11 contains 4 graphics A–D). It is obvious that HI representing 28 countries.

**Fig. 12:**
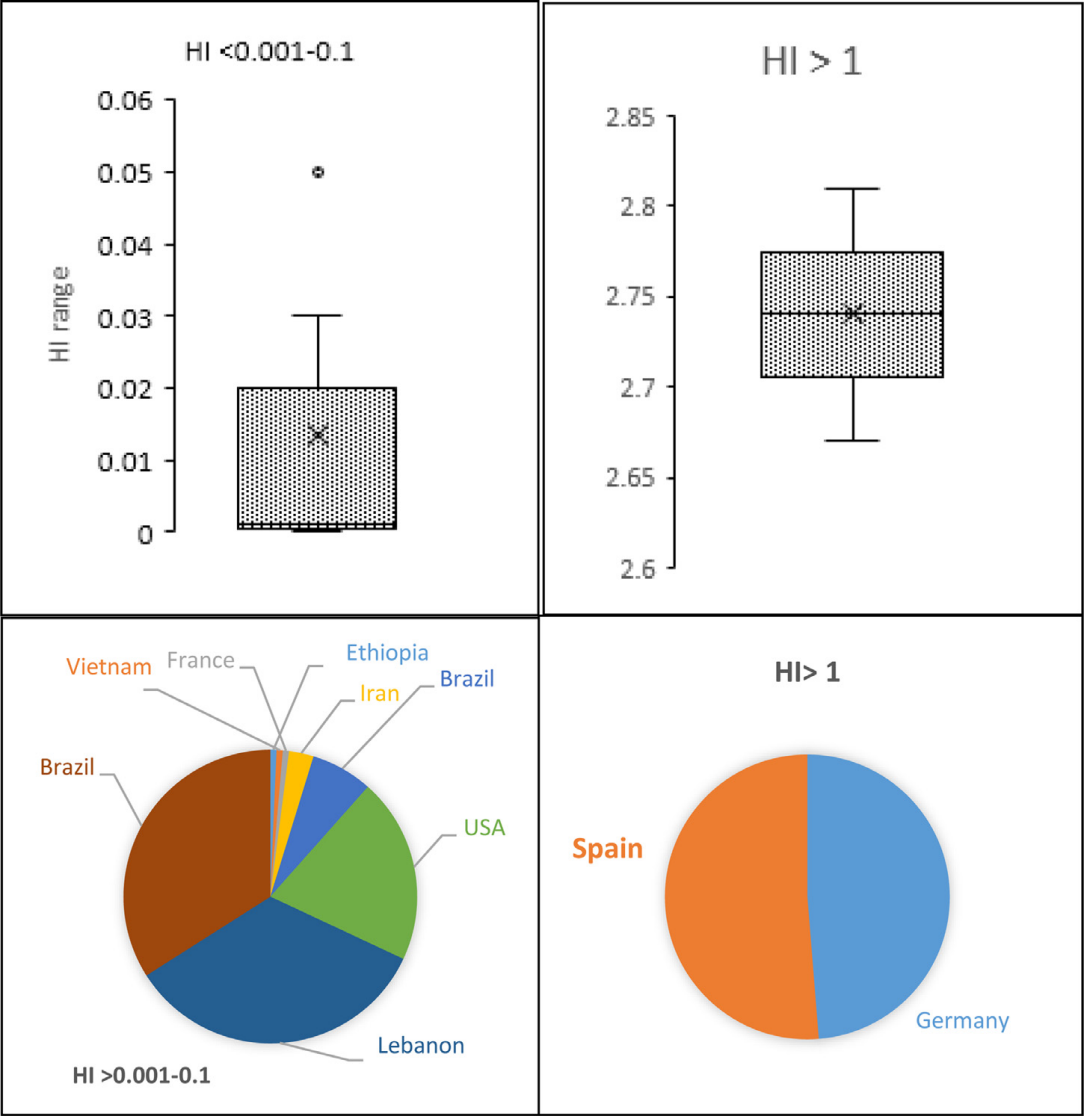
Occurrence of hazard index (HI) associated with HRs in drinking water among several countries. Box plots show the distribution of HI among countries whereas Pie charts show the countries involved in the HI calculation.

Fig. 12 shows HI of herbicide residues as box plot and distribution of countries as Pie chart. It can be seen that two HI categories are shown, HI 0.001–0.1, represent 10 countries whereas H1>1represents two countries. Distribution of countries in the Pie chart shows 13 counties having HI above 0.001 and only two countries having HI above 1, indicating potential risk to humans.

**Fig. 13:**
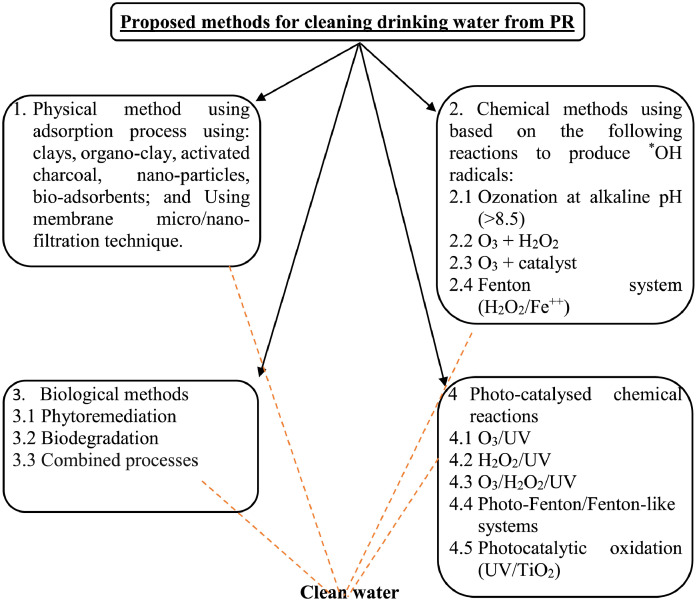
Potential purification methods of PRs from drinking water.

Additionally, the dataset presents several methods with high potential of cleaning water (Fig. 13). It is obvious that the proposed method includes four methods and possible combination such as physical methods, chemical method, biological method and mixed method.

## 2 Experimental Design, Materials and Methods

### 2.1 Data collection

Data were collected using the following specific items

1. Specific phrases “pesticide residues in water, insecticide residues in water; fungicide residues in water, herbicide residues in water”;
2. Chemical name “organochlorine residues in water, organophosphate residues in water, carbamate residues in water, pyrethroid residues in water, neonicotinoid residues in water”,
3. Pesticide name such as “e.g. DDT residues in water, γ-HCH residues in water, Toxaphene residues in water, parathion residues in water, chlorpyrifos residues in water, diazinon residues in water.

### 2.2 Websites used to collect the relevant articles

1 Google engine; 2 Google Scholar; 3 Researchgate; 4 The database of Scopus; 5 Web of Science; 6 Home page of ScienceDirect; 7 Home page of PubMed; 2.2.8 Home page of BMC; 9 Journals home page and 10 direct contact with corresponding authors.

### 2.3 Downloading the articles

Free download articles were collected easily by just a click on the icon, then were saved on our computer. The unfree articles were collected through the university home page or via direct contact with the corresponding author.

### 2.4 Reading and screening the articles

The collected articles were carefully read by the authors. Then, the articles were classified into groups:

Group 1 includes articles developed method to determine pesticide residues in water
Group 2 includes articles determined pesticide residues in agricultural water, rivers, lakes, surface water and ground water
Group 3 includes articles determined pesticide residues in bottled water, and drinking water.

### 2.5 Sorting the articles

The articles were sorted to the following categories:

1. Conference articles. The articles in this section were subdivided into local scientific conference and international scientific conference.
2. Journal articles. The articles in this section are classified according to publishing house into local Journals and International Journals. The international Journals were sorted according to the impact factor and the cite score of the journal. Then were categorized into Q1–Q4 Journals. Articles published in local journals with different publishing house were sorted into Q5, Q6.

### 2.6 Inclusion and exclusion of articles

Articles published in local or international conference with abstracts and/or proceedings were considered irrelevant and excluded. Additionally, articles aimed to develop method for pesticide detection in water resources were also excluded. Moreover, articles which determined pesticide residues in agricultural water, rivers, lakes and wastewater were also excluded. Articles published in a local Journal with multi-disciplines were also excluded.

Inclusion criteria includes articles which determined pesticide residues in drinking water, bottled waters and published in a Journal of Q1-Q4 rank.

### 2.7 Data preparation, modification, calculations and statistical analysis

Pesticide residues in the included articles were collected and inserted to an excel sheet in our computer. The concentrations in all collected articles were normalized to one unit such as µg/L instead of mg/L or ng/L. The latest units were converted to µg/L if found.

### 2.8 Data separation

Pesticide residues were classified into specific groups such as insecticides, herbicides and fungicides. Then, insecticide, herbicide, and fungicide residues were identified and categorized in separate data sheets.

### 2.9 Data processing

Pesticide residues in the included articles were used to calculate country average, standard deviation and/or standard error instead of using the original analyzed data such as minimum, maximum, median, 25th percentile, 75th percentile and/or range.

### 2.10 Calculation of pesticide daily intake (PDI)

PDI associated with drinking water was calculated according to Eq. (1),

(1) [PDI]=([APS]×Q)/BW

where [APR], Q, and BW are the average of pesticide residue found in a drinking water sample (µg/L), the amount of water consumed by a person and body weight (kg), respectively.

It has been shown that drinking water consumption equals 2, 1.5 and 0.75 l for adults, children and infants, respectively, and the BW of adults, children or infants equals 60, 15 and 5 kg, respectively [3].

### 2.11 Calculation of health quotient (HQ) and hazards index (HI)

HQ was calculated according to Eq. (2)

(2) HQ=[PDI]/ARfD

where ARfD is the acute reference dose of pesticide residue expressed in µg/L/day. Value of ARfD was obtained from Ref. [2]. The use of ARfD was previously reported [1,^4^,7].

When a water sample contains more than one pesticide residue, HQ is calculated individually for each sample. HI values equal to/greater than one indicate additive effects and a high risk, whereas values below one indicate low or negligible health risk [4].

(3) HI=HQ1+HQ2+HQ3+HQ4+HQ5+…HQn

When a sample contains only one pesticide residue, then

(4) HI=HQ

In fact, HQ was added in Eq. (3) because a mixture of pesticide residues in which each individual residue is present in the mixture at a level approximating the no observed effect level elicits a measurable response denoted as a joint additive effect. Thus, the summation of individual effects is given in Eq. (3). This is in accordance with  US EPA, [4] and with Tinwell and Ashby [5], who emphasized the joint additive effects of chemical mixtures.

## 3 Statistical Analysis of Pesticide Residues

### 3.1 Classification of pesticide residues according to toxicity class and function

The pesticide residues found in the water samples from a country were subdivided into three groups according to their functions: insecticide, herbicide, and fungicide residues. Each group was subdivided into four toxicity classes (Ia, Ib, II, III and IV) according to Ref. [6].

### 3.2 Calculation of reference average and relative average of pesticide residue

Reference average (Ref Aver) of the insecticide, herbicide or fungicide concentrations (µg/L) was calculated based on Eq. (5).

(5) $$Ref\;Aver=\frac{\left(\left[Ic1\right]+\left[Ic2\right]+\left[Ic3\right]+\ldots \left[Icn\right]\right)}{N}$$

where [Ic1], [Ic2], [Ic3], [Icn], are concentrations of insecticide residue found in country 1, country 2, country 3 and country n (the last country) and N is the total number of insecticide residues found in all countries.

Similarly, reference average of herbicide and fungicide residues was calculated using the corresponding concentrations of herbicides and fungicides and Eq. (5).

Additionally, country average of insecticide concentration was calculated according to Eq. (6)

(6) $$Country\;\;Aver=\frac{\left(\left[I1\right]+\left[I2\right]+\left[I3\right]+\ldots \left[Ix\right]\right)}{Ni}$$

where [*I*1], [*I*2], [*I*3], [*I*x], are concentration of insecticide residue 1, insecticide residue 2, insecticide residue 3 and insecticide residue x and Ni is the total number of insecticide residues found in drinking water in a country.

Similarly, the country average of herbicide and fungicides were calculated.

Then, Rel Aver of insecticide, herbicide and /or fungicide, in each country was calculated according to Eq. (7).

(7) $$Rel\;\;Aver=\frac{Country\;\;average}{Reference\;\;average}$$

Based on the results of Eq. (7), water samples from different countries were subdivided into four categories as follows:

Category I includes countries having Rel Aver value below 0.01, Category II includes countries having Rel Aver values between 0.01 and 0.1, Category III includes countries having Rel Aver values between 0.11 and 0.5; and Category IV includes countries having Rel Aver values ≥ 0.5.

To estimate the significant differences among the Rel Aver values of countries, the reference standard deviation (Ref Stdev) was calculated from the general formula of the calculation. Then, the relative standard deviation of a country was calculated according to Eq. (8)

(8) $$Rel\;\;Stdev=\frac{Country\;\;Stdev}{Reference\;\;Stdev}$$

Then, the Rel Stdev value was used to estimate the relative standard error (Rel Std Error) from Eq. (9) as follows:

(9) $$Rel\;Std\;Error=\frac{Rel\;\;Stdev}{\sqrt[{2}]{n}}$$

where *n* is the number of pesticide residues used to calculate the country Aver. The values of the relative standard errors were used to indicate the error bars in the figures. The overlapping error bars indicate no significant differences in the relative means at *p*-value ≤ 0.05, whereas no overlapping indicates a significant difference in the Rel Aver in the studied countries.

Reference hazard index (Ref HI) was calculated according to Eq. (10)

(10) $$RefHI=\frac{\left(HIc1+HIc2+HIc3+\ldots HIcx\right)}{NC}$$

where HIc1, HIc2, HIc3, HIcx are hazard index in country 1, country 2, country 3 and country x, respectively. NC is the total number of countries.

Rel Hazard index of a country (Rel HIc) can be calculated for Eq. (11)

(11) $$Rel\;HIc\;=\frac{HIc}{Ref\;HI}$$

Application of Eq. (12) enable detection of percentage of differences between Ref aver and Rel Aver. statistical differences between Rel Aver for health hazards and/or concentration averages HIC can be calculated from the relative ration (RR) between any two Rel HI using Eq. (12)

(12) $$\%\;Difference\;=1-\;\frac{\left(Ref\;Aver-Country\;Aver\right)}{Ref\;Aver}\;$$

Values of% difference < 0.05 indicate significant differences.

Furthermore, the values of Rel Aver, of all countries were used to draw forest plot. Relative standard error and Relative standard deviation were taken as lower and upper limit of error bars in the forest plot. Then perpendicular axe was placed in certain points to show the differences.

Additionally, box plot was drawn for each of insecticide and herbicide residues to show the distribution of insecticide and herbicide in each country.

## Ethics Statements

This work does not involve human subjects, animal experiments, and/or data collected from social media platforms.

## CRediT authorship contribution statement

**Ibrahim El-Nahhal:** Data curation, Writing – original draft. **Yasser El-Nahhal:** Supervision, Visualization, Formal analysis, Writing – review & editing.

## Declaration of Competing Interest

The authors declare that they have no known competing financial interests or personal relationships that could have appeared to influence the work reported in this paper.
